# Compartmentation and channelling of metabolites in the human cell line AGE1.HN^®^

**DOI:** 10.1186/1753-6561-5-S8-P84

**Published:** 2011-11-22

**Authors:** Jens Niklas, Volker Sandig, Elmar Heinzle

**Affiliations:** 1 Biochemical Engineering Institute, Saarland University, 66123 Saarbrücken, Germany; 2 ProBioGen AG, 13086 Berlin, Germany

## Background

A thorough knowledge of the metabolism and its compartmentation in mammalian cells is desirable to enable rational design and optimization of producing cell lines and production processes for biopharmaceuticals. In this study we focused on acquiring a detailed understanding of metabolite channelling and the metabolic flux distribution during overflow metabolism in the human cell line AGE1.HN^®^ (ProBioGen AG). This metabolic phenotype characterized by energy spilling as well as waste product formation is commonly observed in the beginning of the cultivation [1]. ^13^C tracer experiments and ^13^C flux analysis as applied in this investigation represent methods offering in-depth insights into cellular physiology [2,3].

## Materials and methods

### Cultivation and analysis

The human neuronal cell line AGE1.HN^®^ (ProBioGen AG, Berlin, Germany) was used. Cultivations were carried out in shake flasks (Corning, NY, USA) or bioreactor filter tubes (TPP, Trasadingen, Switzerland). ^13^C-labeling experiments using the tracers [1,2-^13^C_2_] glucose , [U-^13^C_5_] glutamine, [U-^13^C_3_] alanine, [1-^13^C_1_] lactate (Cambridge Isotope Laboratories, Andover, MA, USA) and [U-^13^C_6_] glucose (Euriso-Top, Saarbrücken, Germany) were conducted. Extracellular metabolites were quantified using different HPLC methods [4,5]. ^13^C-labeling of metabolites was analysed using GC-MS [6]. Carbon mass isotopomers were determined from the analyte mass isotopomer distribution [7].

### Carbon atom transition model

A carbon atom transition model was set up using the Kyoto Encyclopedia of Genes and Genomes (www.kegg.com) pathway database for *Homo sapiens*.

### ^13^C metabolic flux analysis

Fluxes were estimated using the method of Yang et al. [8] applying Matlab R2008 (The Mathworks, Natick, MA, USA).

## Results

Experiments applying ^13^C-labelled glucose, glutamine, alanine and lactate tracers were carried out to identify active pathways and channelling of metabolite carbons in the central metabolism of AGE1.HN^®^. It was observed that almost 80% of glucose consumed was detected in lactate. Smaller amounts were channeled to alanine (5%) and serine (3%). Glucose carbons were additionally entering the tricarboxylic acid (TCA) cycle which can be deduced from an increase in fractional labelling of glutamate and proline in glucose tracer experiments. Reflux from TCA cycle metabolites to glycolytic metabolites was also detected since labelling in lactate as well as alanine was measured when using [U-^13^C_5_] glutamine as tracer. Decrease in labelled extracellular lactate as well as an increase in labelled extracellular alanine as observed in the lactate tracer experiment shows that lactate was not only produced but also taken up during overflow metabolism. Furthermore, the lactate and alanine tracer experiments indicated that alanine and lactate and subsequently the pyruvate pools used for their synthesis are connected. Alanine taken up was mainly transaminated entering the cytosolic pyruvate pool, converted to lactate and secreted.

Fluxes were calculated for the growth phase between day 1 and day 4 of the cultivation in which the cells exhibit overflow metabolism characterized by production of waste metabolites [1].

For flux calculation extracellular and anabolic rates as well as the labelling information stored in extracellular lactate resulting from three different parallel tracer experiments using [1,2-^13^C_2_] glucose, [U-^13^C_6_] glucose and [U-^13^C_5_] glutamine was included. In theory one could also use the labelling information stored in the building blocks of the cellular macromolecules, namely proteins, carbohydrates, lipids or nucleic acids as it is widely applied in microbial ^13^C flux analysis. However, the time that is needed to incorporate the labelling in these molecules as well as the presence of huge amounts of unlabelled species caused by high seeded cell densities needed in mammalian cell culture processes is a huge disadvantage. Another possibility would be using the labelling patterns of intracellular metabolites. This requires, however, sampling methods that are still not well established in mammalian suspension cell culture. A main problem represents hereby the quenching procedure that remains still a huge challenge for suspension cell culture and it cannot be guaranteed that the measured labelling or intracellular concentration is really representing intracellular values not corrupted with extracellular species. The labelling of extracellular amino acids, which could also be used theoretically, is however not directly representing the labelling of intracellular species. This is caused by the fact that the amino acids are provided in the medium and the reversibility of production and uptake of these and their conversion dilutes the labelling which is then not representing the labelling of metabolites in central metabolism of the cell. This could only be solved by detailed knowledge and modelling of reaction reversibilities. In this study solely lactate labelling was therefore included in the metabolic flux estimation since it is not present in the beginning of the cultivation representing at steady state directly the labelling of cytosolic pyruvate which is probably the most important metabolic hub in the cell.

It was observed that the flux through the oxidative branch of the pentose phosphate pathway was very low being around 2% of the glycolytic flux which might be compensated in AGE1.HN^®^ by a high flux through malic enzyme additionally producing NADPH. The cytosolic pyruvate pool was fed mainly by the glycolytic flux, however, pyruvate taken up was also significantly contributing to intracellular pyruvate. Cytosolic pyruvate was almost exclusively converted to lactate and alanine and secreted. Flux between cytosolic oxaloacetate and cytosolic pyruvate was found to be reversible with similar fluxes in both directions. Pyruvate transport between mitochondria and cytosol was very low. Mitochondrial oxaloacetate pool was fed by cytosolic oxaloacetate. The flux from mitochondrial oxaloacetate to mitochondrial pyruvate was relatively high. The tricarboxylic acid cycle was fed mainly via α-ketoglutarate and oxaloacetate.

## Conclusions

In order to improve metabolic efficiency in AGE1.HN^®^ engineering strategies focussing on improved metabolite transfer between cytosolic and mitochondrial pyruvate pools could be applied accompanied by improved control and targeted reduction of substrate availability and uptake. The ^13^C flux analysis method that was applied in this study allows a detailed determination of metabolic fluxes in the central metabolism of mammalian cells and can thus be applied for studying related biological questions. The presented data is an important step for an improved system level understanding of the AGE1.HN cell line.
